# Cell cycle activation in thyroid hormone-induced apoptosis and stem cell development during *Xenopus* intestinal metamorphosis

**DOI:** 10.3389/fendo.2023.1184013

**Published:** 2023-05-17

**Authors:** Yuta Tanizaki, Yuki Shibata, Wonho Na, Yun-Bo Shi

**Affiliations:** Section on Molecular Morphogenesis, Eunice Kennedy Shriver National Institute of Child Health and Human Development (NICHD), National Institutes of Health (NIH), Bethesda, MD, United States

**Keywords:** programmed cell death, metamorphosis, *Xenopus laevis*, *Xenopus tropicalis*, postembryonic development, intestine, thyroid hormone receptor, stem cell

## Abstract

Amphibian metamorphosis resembles mammalian postembryonic development, a period around birth when many organs mature into their adult forms and when plasma thyroid hormone (T3) concentration peaks. T3 plays a causative role for amphibian metamorphosis. This and its independence from maternal influence make metamorphosis of amphibians, particularly anurans such as pseudo-tetraploid *Xenopus laevis* and its highly related diploid species *Xenopus tropicalis*, an excellent model to investigate how T3 regulates adult organ development. Studies on intestinal remodeling, a process that involves degeneration of larval epithelium via apoptosis and *de novo* formation of adult stem cells followed by their proliferation and differentiation to form the adult epithelium, have revealed important molecular insights on T3 regulation of cell fate during development. Here, we review some evidence suggesting that T3-induced activation of cell cycle program is important for T3-induced larval epithelial cell death and *de novo* formation of adult intestinal stem cells.

## Introduction

**Figure 1:**
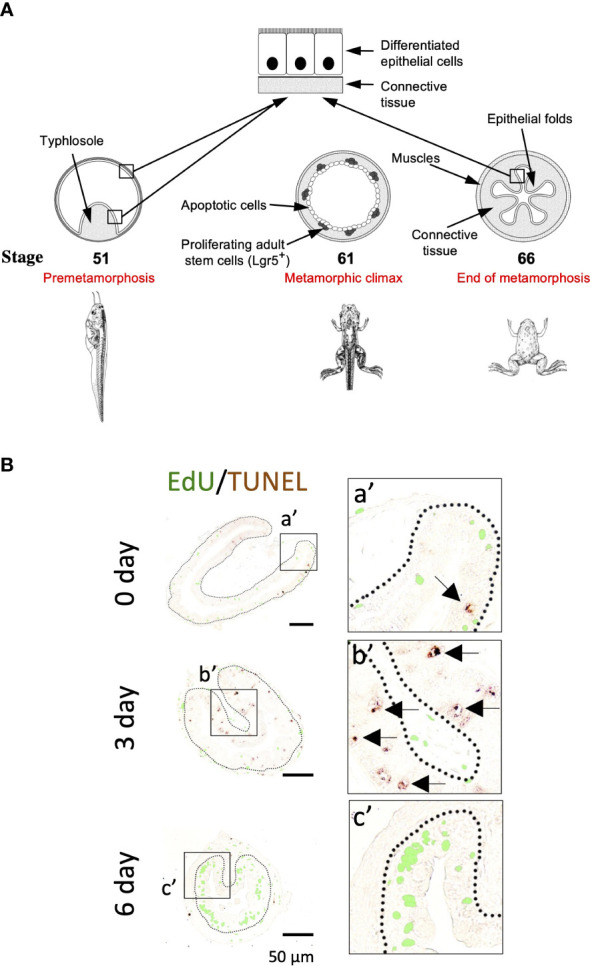
**(A)**. Schematic diagram of *Xenopus* intestinal metamorphosis. Both tadpole and frog intestine are structurally simple, consisting of mainly three tissue layers: inner epithelium, connective tissue, and outer muscle layers. The tadpole intestine is much simpler, with only a single epithelial fold, the typhlosole. In contrast, the frog intestine has multiple epithelial folds with elaborate connective tissue and muscle layers. The major events underlying the change from tadpole to frog intestine during metamorphosis include the apoptosis of essentially all larval epithelial cells, as indicated by circles. Concurrently, the adult epithelial stem cells, with high level expression of known stem cell markers such as Lgr5, are formed *de novo* through dedifferentiation of some larval epithelial cells and rapidly proliferate at the climax metamorphosis, as indicated by the dots. The connective tissue and muscle cells also develop extensively during metamorphosis. **(B)**. Apoptotic and proliferating cells are non-overlapping epithelial cells during T3-induced intestinal metamorphosis. Premetamorphic *Xenopus laevis* tadpoles at stage 54 were treated with 10 nM T3 for 0, 3, or 6 days and sacrificed one hour after injection with EdU to label proliferating cells. Intestinal cross-sections were double stained for EdU and by TUNEL for apoptotic cells. A higher magnification of the boxed areas labeled with a’-c’ is shown on the right. The dotted lines depict the epithelium-mesenchyme boundary. Note that apoptosis as shown by the TUNEL signal in the epithelium occurred prior to the appearance of the clusters (islets) of EdU-labeled cells and in distinct epithelial cells during T3 treatment (c’). Arrows point to some apoptotic cells. See (11) for more details.

The development of vertebrate intestine, like many other organs, takes place in two phases, the initial formation of a neonatal/juvenile form and subsequent maturation into the adult form. This second phase often occurs during postembryonic development, a perinatal period when plasma thyroid hormone (T3) level peaks (1–3). This period corresponds the first 2-3 weeks after birth in mouse and metamorphosis in amphibians such as the highly related anurans pseudo-tetraploid *Xenopus laevis* and diploid *Xenopus tropicalis* (Note that due to the conservations between the two species, we will simply refer to both as *Xenopus* unless specified, although earlier studies on anuran metamorphosis were mainly on *Xenopus laevis* while more recent ones, particularly gene knockout studies, have been on *Xenopus tropicalis*). Importantly, maturation of the intestine during this second phase appears to be highly conserved (4–10). For example, the mouse intestine has villi but no crypts, where adult stem cells reside, at birth and develop crypts during the first 3 weeks after birth when T3 levels are high. Similarly, the intestine in a premetamorphic *Xenopus* tadpole, when there is little or no T3, is also simple in structure, consisting of mostly a single layer of epithelial cells, surrounded by thin layers of connective tissue and muscles (**Figure 1A**) (12–17). As T3 levels rises after stage 54 (about 4 weeks of age) (18, 19), metamorphosis begins and larval epithelial cells undergo programmed cell death (12, 13, 20). Some larval epithelial cells undergo dedifferentiation during metamorphosis to form clusters of cells that proliferate rapidly and express well-known adult intestinal stem cell markers such as Lgr5 by climax of metamorphosis, e.g., stage 61 (about 6-7 weeks of age) (**Figure 1A**) (11, 21, 22). By the end of metamorphosis or stage 66 (about 2 months after fertilization), these proliferating stem cells differentiate to form a multi-folded epithelium surrounded by elaborate connective tissue and muscles (4, 16, 17, 23, 24). In the adult frog, the intestinal stem cells are localized at the bottom of the epithelial fold while cell death occurs mainly at the crest of the fold, similar to those taking place in the crypt-villus unit in adult mammalian intestine (16, 25).

T3 not only has peak levels during postembryonic development but also plays critical roles during this period, with T3 deficiency causing severely developmental problems in all vertebrates including human (1, 2, 26, 27). T3 is both necessary and sufficient for anuran metamorphosis. Thus, preventing the synthesis of endogenous T3 allows *Xenopus* tadpoles to remain in tadpole form for years while wild type animals typically finish metamorphosis by around 2 months of age (1, 2, 15). Conversely, treating premetamorphic *Xenopus* tadpoles with physiological levels of T3 in the rearing water causes precociously metamorphosis. Making use of the ability to easily manipulate anuran metamorphosis by controlling the availability of T3 to tadpoles or even organ or primary cell cultures and the advancement in genetic technologies, especially gene-editing for knockout studies in the diploid *Xenopus tropicalis* (28–35), we and others have been studying the molecular mechanism by which T3 regulates cell fate and tissue transformation during metamorphosis. Here, we review some recent studies on intestinal remodeling, with an emphasis on the potential role of cell cycle activation in larval epithelial cell death and adult stem cell development.

## T3 induces larval epithelial cell death and adult stem cell development in an organ autonomous manner via T3 receptor

Intestinal remodeling, just like any other events during metamorphosis, requires T3. Treatment of premetamorphic *Xenopus* tadpoles with T3 leads to premature intestinal metamorphosis (16). The most noticeable changes during intestinal metamorphosis are the nearly 90% reduction in the length of the small intestine, while the most dramatic tissue transformation occurs in the epithelium with the larva epithelial cells induced to undergo apoptosis by T3, followed by rapid proliferation of newly formed cell clusters in the epithelium (**Figure 1B**) (11). Importantly, these proliferating cells express high levels of known markers of adult mammalian intestinal stem cells such as Lgr5 (**Figure 1B**) (11), suggesting that T3 induces the formation of adult stem cells during metamorphosis.

Importantly, T3-treatment of intestinal organ cultures from premetamorphic tadpoles also leads to larval epithelial cell death and *de novo* formation of adult stem cells, indicating that the adult stem cells develop organ-autonomously in an T3-dependent manner. More importantly, by using recombinant organ cultures generated from isolated intestinal epithelium and non-epithelial tissues (the rest of the intestine after separating the epithelium) from premetamorphic wild type tadpoles and transgenic tadpoles expressing GFP ubiquitously, we have shown that T3-induced stem cells originate from larval epithelium, likely due to dedifferentiation of some larval epithelial cells (23) since there has been no evidence of pre-existing epithelial stem cells in the tadpole intestine and that the differentiated larval epithelial cells are capable of proliferation (16, 36). These findings also indicate that T3 is both necessary and sufficient for larval intestinal cell death and adult stem cell development during metamorphosis.

T3 functions mainly by binding to T3 receptors (TRs) to regulate target gene transcription (37–40). There are two types of TR genes, TRα and TRβ, in all vertebrates. TRs can activate or repress target gene transcription in the presence or absence of T3, respectively, by binding, as heterodimers with 9-cis retinoic acid receptors (RXRs), to specific DNA sequences called T3-response elements (TREs) within target genes (41–45). Earlier studies on the expression patterns and molecular properties of TRs in *Xenopus laevis* have led to a dual function model for TRs during *Xenopus* metamorphosis (46). According to the model, TRs are mainly unliganded in premetamorphic tadpoles when there is little or no T3 and thus repress target genes to prevent precocious metamorphosis. During metamorphosis when T3 level is high, T3 binds to TR to activate target genes, thus leading to tadpole metamorphosis. Extensive molecular and transgenic studies in *Xenopus laevis* and gene knockout studies in *Xenopus tropicalis* have provided strong support for this model (47–58).

A critical role of TR in intestinal metamorphosis was demonstrated by studies with recombinant organ cultures made of intestinal epithelium and non-epithelial tissues from premetamorphic wild type tadpoles or transgenic ones containing a heat shock-inducible dominant positive TR (dpTR) that cannot bind to T3 but functions like a constitutively liganded TR (59, 60). In these recombinant organ cultures, T3 signaling can be activated in either the epithelium or non-epithelial tissues or both by heat shock treatment of the organ cultures without the presence of T3. Such studies have revealed that dpTR expression in both epithelium and non-epithelium can induce intestinal metamorphosis, including larval epithelial apoptosis and adult intestinal stem cell formation and their subsequent proliferation and differentiation, in the absence of T3 (60). These findings indicate that TR is sufficient for mediating all effects of T3 for intestinal metamorphosis, including larval epithelial cell death and adult stem cell development. Interestingly, activating T3-signaling by expressing dpTR in either the epithelium or non-epithelial tissues alone can induce larval epithelial degeneration, indicating that larval epithelial apoptosis can be induced by T3-signaling both cell autonomously and via cell-cell interaction. However, dpTR expression in either the epithelium or the non-epithelial tissues alone fails to induce the formation of adult stem cells, although dpTR expression in the epithelium alone results in dedifferentiation of some larval epithelial cells (60). These findings suggest that epithelial T3-signaling induces larval epithelial cell dedifferentiation while T3-signaling in the non-epithelial tissues is required to help such dedifferentiated cells to develop into stem cells, likely via the formation of a proper stem cell niche through cell-cell and/or cell-ECM (extracellular matrix) interactions (60–63).

## TR is not needed for adult intestinal morphogenesis but is essential for larval epithelial cell death and adult intestinal stem cell development during metamorphosis

The role of endogenous TR in regulating intestinal metamorphosis was first suggested by transgenic studies with dominant negative mutant TRs that cannot bind to T3. The expression of such dominant negative TRs was found to inhibits *Xenopus laevis* metamorphosis, including intestinal remodeling (50, 61, 64, 65). Since the dominant negative TRs compete functionally against endogenous wild type TR that can bind T3, the findings are not surprising given the causative role of T3 in all aspects of metamorphosis but demonstrate an essential role for TR to mediate the metamorphic effects of T3, including larval intestinal cell death and adult stem cell development.

With the advancement of gene editing technologies, it became possible to knock out endogenous TR genes in *Xenopus.* Indeed, individual TRα and TRβ genes or both have been knocked out recently in the diploid *Xenopus tropicalis* and found to have distinct tissue-dependent effects during metamorphosis (52, 55–58, 66–70). Consistent with the high but relatively constant expression of TRα during intestinal metamorphosis (71), knocking out TRα delayed intestinal remodeling (66, 69, 72). Surprisingly, knocking out TRβ had relatively subtle effect on intestinal remodeling during natural metamorphosis (52, 55), although TRβ expression, which is very low in premetamorphic tadpole intestine, is dramatically upregulated during intestinal metamorphosis (71). On the other hand, when premetamorphic wild type and TRβ knockout tadpoles were induced to metamorphose with exogenous T3 treatment, both larval epithelial cell death and adult intestinal stem cell formation, which occurred within 2-3 days in wild type tadpoles after the treatment, were delayed or inhibited in the TRβ knockout tadpoles (55). These findings suggest that TRα and TRβ have distinct but compensatory roles during intestinal metamorphosis. As T3-induced metamorphosis occurs much faster than the 2-3 weeks required for the premetamorphic tadpoles at stage 54 to develop to the climax (stages 60-62, when larval cell death and adult epithelial stem cell formation occur) during the natural metamorphosis, the compensation by TRα may be too slow to prevent the effects of TRβ knockout on intestinal remodeling during T3-induced metamorphosis but fast enough to prevent any major defect in intestinal remodeling during natural metamorphosis in TRβ knockout animals.

**Figure 2:**
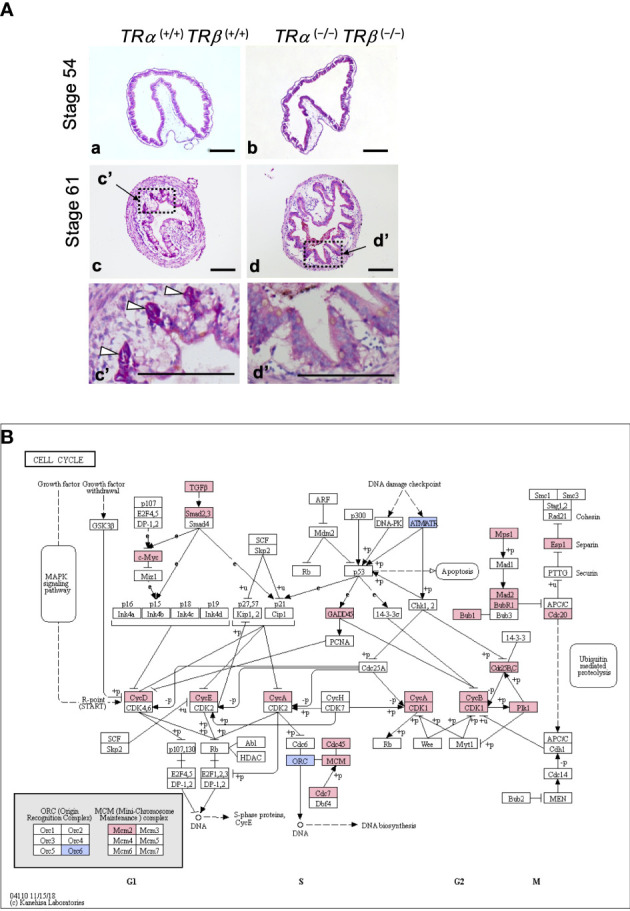
**(A)** TR double knockout tadpoles have abnormal intestinal morphology with premature adult type epithelial folding. Cross-sections of the intestine of indicated genotypes stages were stained with methyl green-pyronin Y. (a, c): Wild type *TRα ^(+/+)^TRβ ^(+/+)^*; and (b, d): TR double knockout *TRα ^(−/−)^TRβ ^(−/−)^*. Dashed boxes in c and d are shown in higher magnification in c’ and d’, respectively. White arrowheads point to the clusters of proliferating adult epithelial stem cells adjacent to/underneath the degenerating larval epithelium (vacuole-like, poorly stained) at the climax of metamorphosis (stage 61) in wild type tadpoles. Note that the knockout tadpoles lacked such clusters at stage 61 and the epithelium appeared to be uniform without any obvious degeneration, but with numerous folds. Bars: 100 μm. See (73) for details. **(B)** TRα is required for the activation of many cell cycle genes during early phase of T3-induced intestinal remodeling. Genes regulated by at least 2.0-fold after 18 hours of T3 treatment in stage 54 wild type but not TRα knockout tadpoles were mapped onto the KEGG pathway for cell cycle. Pink boxes indicate upregulation and blue boxes indicate downregulation. Note that most of the regulated genes were upregulated by T3. See (72) for more details.

When both TRα and TRβ genes were knocked out in *Xenopus tropicalis*, the tadpoles could develop to the climax stage 61 and died after about 2 weeks at stage 61, in contrast to wild type tadpoles that develop from stage 61 to the end of metamorphosis in a week (56). While the wild type intestine at stage 61 had extensive larval epithelial cell death and proliferation of new formed adult epithelial stem cells, TR double knockout tadpoles at stage 61 had little larval cell death or adult stem cell proliferation (**Figure 2A**) (73). In addition, as predicted, T3 treatment of premetamorphic TR double knockout animals had no effect on the intestine (56, 73), in contrast to the wild type animals (**Figure 1B**). Surprisingly, the intestine of TR double knockout tadpoles developed adult morphology (with numerous epithelial folds and thick layers of connective tissue and muscles) precociously, by as early as stage 58, mimicking the wild type intestine at the end of metamorphosis (stage 66) (**Figure 2A**) (73). These findings indicate that TR is required for T3-induced larval epithelial cell death and adult epithelial stem cell development but not for adult intestinal morphogenesis during metamorphosis.

## Cell cycle activation by liganded TR is involved in larval epithelial cell death and adult stem cell development

As a transcription factor, TR regulates target gene transcription in a T3-dependent manner. Toward understanding how T3 regulates intestinal remodeling, various approaches have been used to isolate and characterize T3-response genes during *Xenopus* intestinal metamorphosis (74–78). These studies have revealed, perhaps expectedly, that many genes in signaling pathways known to be important for stem cell proliferation and function are induced by T3 during intestinal remodeling. These pathways include hedgehog pathway (21, 79–82), Wnt signaling (83–85), Notch pathway (86), and BMP signaling (87, 88), etc. In addition, many other genes, such as the methyl-CpG binding domain protein 3 (MBD3) (89) and tRNA methyltransferase-like 1 (Mettl1) (90), that were not previously known to associated with cell death or stem cells, were found to be highly upregulated by T3 during intestinal metamorphosis, suggesting that they may be novel regulators of cell death or stem cells during development.

When global gene expression analyses with RNA-seq were carried out on wild type and TR knockout intestine during metamorphosis, it was revealed that many more genes in gene ontology (GO) terms related to stem cells, cell proliferation, and apoptosis were upregulated in the wild type tadpole intestine at the climax of metamorphosis (stage 61) compared to premetamorphic stage 54 than in the TR double knockout intestine (73), consistent with the massive larval epithelial cell death and formation/proliferation of adult stem cells at the climax of metamorphosis in the wild type but not TR double knockout intestine. In addition, GO and KEGG pathway analyses of the genes that were regulated between stage 54 and stage 61 in the intestine of wild type and TR double knockout animals showed that many GO terms and KEGG pathways, particularly cell cycle/proliferation-related ones, were enriched among the upregulated genes in the wild type but not TR double knockout intestine. In fact, several cell cycle/proliferation-related GO terms were even enriched among genes downregulated at stage 61 compared to stage 54 in the TR double knockout intestine. Furthermore, when GO and KEGG pathway analyses were performed on the genes expressed at higher levels in the intestine of wild type than TR double knockout tadpoles at stage 61, it was again found that many cell cycle-related GO terms/pathways were enriched among these genes (73). These findings suggest that T3-bound TR activates cell cycle programs to facilitate intestinal metamorphosis. Given the rapid proliferation of adult stem cells at the climax of intestinal metamorphosis in the wild type (**Figure 1**) but not TR double knockout tadpoles, it is not surprising to find the upregulation of genes in the cell cycle program in the wild type intestine compared to the TR double knockout ones at the climax of metamorphosis.

A recent RNA-seq analysis of intestinal gene expression in premetamorphic wild type and TRα knockout tadpoles at stage 54 after 18 hours of T3 treatment suggests that activation of cell cycle program may also be important for T3-induced larval epithelial cell death during metamorphosis (72). During T3-induced metamorphosis, larval epithelial cell death is induced dramatically after 2 days while epithelial cell proliferation is increased significantly only after 3 days, due to the formation of adult epithelial stem cells (**Figure 1**) (11, 91). Thus, it was expected that T3 would not induce cell proliferation program, e.g., cell cycle activation, in the first 2 days of treatment. Surprisingly, GO and KEGG pathway analyses of the genes regulated by 18 hour T3 treatment of wild type tadpoles showed significant enrichment of GO terms and KEGG pathways related to cell cycle (72). This raises a possibility that the activation of the cell cycle program by T3 is an important early step for T3 to induced larval epithelial cell death (11, 72, 91). Furthermore, when the gene expression in the intestine of wild type and TRα knockout tadpoles with or without 18 hour T3-treatment were compared, it was found that the endogenous TRα was important for the regulation of the cell cycle program (72), including the KEGG cell cycle pathway, where many genes were upregulated by T3 in the wild type but not TRα knockout tadpoles (**Figure 2B**). This important role of TRα in gene regulation by T3 during the early phase of T3-induced metamorphosis is consistent with the fact that there is little TRβ expression in premetamorphic tadpole intestine (71). Thus, T3 likely activates the cell cycle program via TRα early during metamorphosis to facilitate epithelial cell fate determination in the intestine: apoptosis *vs*. dedifferentiation into adult stem cells.

Whether cell cycle activation leads to larval epithelial cell death and adult stem cell development remains to be elucidated. As cell cycle activation is typically associated with cell proliferation in development, the discovery of a role of cell cycle activation in developmental cell death was surprising. On the other hand, there has been evidence for involvement of cell cycle regulators in apoptosis. For example, c-Myc, a well-known oncogene that activate target gene transcription and promote cell proliferation, can induce cell death when overexpressed, at least in cell cultures (92–102). It is possible that that over activation of cell cycle pathways may lead to apoptosis in some cells. As the larval intestinal epithelial cells are mitotically active, even though differentiated sufficiently to function in the tadpole (16), T3-induced further activation of the cell cycle/proliferation pathways in such mitotically active yet differentiated cells may force to them to change their fate, either death via apoptosis or dedifferentiation to become adult stem cells to accommodate the faster proliferation needed for intestinal metamorphosis. Such a mechanism may explain why cell cycle/proliferation pathways are activated in the larval epithelium prior to and throughout the two major epithelial transformations, apoptotic larval epithelial degeneration, and *de novo* formation of the adult epithelium, during T3 intestinal remodeling.

## Conclusion

The ability of vertebrate intestinal epithelium for self-renewal throughout adulthood has made the intestine a well-studied model for analyses of the properties and regulation of adult organ-specific stem cells (4–10). The formation of the adult intestinal stem cells during vertebrate development is much less known but appears to be conserved in vertebrates. In both mouse and *Xenopus*, the formation of the adult intestinal stem cells occurs during postembryonic development (the neonatal period in mouse and metamorphosis in *Xenopus*) when plasma T3 level peaks, and T3-signaling is important for the development and/or maintenance of the adult intestine (6, 103–111). These suggest a conservation in T3-regulation of adult intestinal development.

Studies on intestinal metamorphosis in *Xenopus laevis* and *Xenopus tropicalis* have revealed important and novel insights on how T3 regulates the development of the adult intestine. First, T3 induces *de novo* formation of adult intestinal epithelial stem cells via larval epithelial cell dedifferentiation. Second, T3-signaling in both the epithelium and non-epithelial tissues are required for adult stem cell development, likely involving the formation of adult stem cell niche. Third, TR is essential to mediate T3 signaling for both larval epithelial cell death and the formation of adult stem cells while adult intestinal morphogenesis does not require TR. Finally, and importantly, global gene expression studies on intestinal development in wild type and TR knockout tadpoles have not only revealed the regulation of diverse GO terms and pathways by T3 during intestinal metamorphosis but also implicate a surprising and novel role of cell cycle activation by T3 in cell fate determination. It would be interesting to test whether the activation of cell cycle by T3 is indeed a prerequisite for larval epithelial cells to choose between apoptosis or dedifferentiation into stem cells during intestinal metamorphosis. Future studies on the potentially conserved functions of the T3-induced GO terms and pathways in the development of the adult intestine in other species should improve our understanding of the development and function of adult stem cells in human intestinal homeostasis and diseases.

## Author contributions

All authors contributed to the article and approved the submitted version.
